# Legacy effects of short-term intentional weight loss on total body and thigh composition in overweight and obese older adults

**DOI:** 10.1038/nutd.2016.8

**Published:** 2016-04-04

**Authors:** E A Chmelo, D P Beavers, M F Lyles, A P Marsh, B J Nicklas, K M Beavers

**Affiliations:** 1 J Paul Sticht Center on Aging, Section on Gerontology and Geriatric Medicine, Department of Internal Medicine, Wake Forest School of Medicine, Winston-Salem, NC, USA; 2 Department of Biostatistical Sciences, Wake Forest School of Medicine, Winston-Salem, NC, USA; 3 Department of Health and Exercise Science, Wake Forest University, Winston-Salem, NC, USA

## Abstract

**Objective:**

Weight regain following intentional weight loss may negatively impact body composition, accelerating fat regain and increasing risk of physical disability. The purpose of this study was to compare long-term changes in whole body and thigh composition in obese older adults who intentionally lost and then partially regained weight to obese older adults who remained weight stable.

**Subjects/Methods:**

This pilot study analyzed total body (dual-energy X-ray absorptiometry (DXA)) and thigh (computed tomography (CT)) composition data collected from 24 older (65–79 years) adults 18 months after completion of a 5-month randomized trial that compared resistance training alone (RT) with RT plus caloric restriction (RT+CR).

**Results:**

Mean loss of body mass in the RT+CR group (*n=*13) was 7.1±2.4 kg during the 5-month intervention (74% fat mass; 26% lean mass; all *P<*0.01), whereas RT (*n=*11) remained weight stable (+0.3±1.8 kg; *P=*0.64). Differential group effects were observed for all DXA and CT body composition measures at 5 months (all *P*⩽0.01); however, by 23 months, group differences persisted only for total body (RT+CR: 81.6±10.0 kg vs RT: 88.5±14.9 kg; *P=*0.03) and lean (RT+CR: 50.8±9.3 kg vs RT: 54.4±12.0 kg; *P<*0.01) mass. All RT+CR participants regained weight from 5 to 23 months (mean gain=+4.8±2.6 kg; *P<*0.01). Total fat mass and all thigh fat volumes increased, whereas thigh muscle volume decreased, during the postintervention follow-up in RT+CR (all *P*⩽0.01). In the RT group, body mass did not change from 5 to 23 months (−0.2±0.9 kg; *P=*0.87). Decreased total thigh volume, driven by the loss of thigh muscle volume, were the only postintervention body composition changes observed in the RT group (both *P<*0.04).

**Conclusions:**

Short-term body composition benefits of an RT+CR intervention may be lost within 18 months after completion of the intervention.

## Introduction

Obesity and aging are major risk factors for the development and recurrence of a wide array of chronic disease.^1, 2^ In addition, aging is associated with increased adiposity, including preferential fat deposition in ectopic regions,^3^ such as within the skeletal muscle.^4^ The amount of fat stored in and around thigh muscle, especially intermuscular fat, is associated with reduced muscle function and strength.^5, 6, 7^ When coupled with the well-documented age-related loss of muscle mass (e.g., sarcopenic obesity), these changes in body composition may accelerate declines in physical function and increase risk of physical disability.^8^

Lifestyle-based weight loss interventions in obese older adults, including caloric restriction and exercise, can be very effective in reducing total body and ectopic fat stores, at least in the short term. However, weight regain following intentional weight loss is common,^12^ and emerging evidence suggests that it may negatively influence body composition in older adults and perhaps promote sarcopenic obesity. As we^13, 14^ and others^15, 16^ have shown, older adults show a faster accumulation of total fat vs muscle mass during weight regain after intentional weight loss. Observational data of older adults also show that proportionally more muscle mass is lost during periods of weight loss than is gained during periods of weight gain or regain.^17, 18, 19^ However, these prior studies do not discriminate body composition changes because of a cycle of weight loss and regain from longitudinal aging-related changes. The ‘lack of adjustment for the age-related decline in fat-free mass' is cited as a major limitation to full understanding of the ramifications of weight cycling on body composition in older adults.^20^ As this population is also more likely to store excess energy as fat in ectopic regions,^4, 21, 22, 23^ it is also important to determine whether weight regain following weight loss results in increased relative fat storage in these less desirable locations.

Thus, the purpose of this study was to compare whole body and thigh composition in older adults who intentionally lost and then partially regained weight to older adults who remained weight stable. We accomplished this by recalling participants 18 months after completion of a 5-month randomized trial designed to compare a resistance training and caloric restriction (RT+CR) intervention to resistance training alone (RT). We also compared changes in whole body and thigh composition within groups, highlighting changes that occurred with weight loss and subsequent weight regain.

## Materials and methods

### Study overview

This pilot study collected and analyzed whole-body and thigh composition data 18 months after completion of a 5-month randomized controlled trial, originally designed to compare the effects of RT+CR to RT alone on change in body composition and skeletal muscle function. The parent study design, recruitment and main outcome data are published.^11^ Briefly, men and women were recruited and enrolled in the parent study on the basis of the following criteria: (a) age 65–79 years, (b) no resistance training in the past 6 months, (c) body mass index=27–34.9 kg m^−^^2^, (d) non-smoking for the past year, (e) weight stable (<5% weight change), (f) normal cognitive function and, (g) no evidence of clinical depression, heart disease, cancer, liver or renal disease, chronic pulmonary disease, uncontrolled hypertension, physical impairment or other contraindication for exercise.

After completion of the 5-month trial, a subsample of participants in the RT+CR group who lost ⩾5% of their body weight during the intervention (*n=*13) and participants in the RT group who lost <5% of their body weight (*n=*11) and who had complete DXA and CT measurements were contacted sequentially until we reached our *a priori* enrollment sample size (*n=*24). This study was approved by the Wake Forest School of Medicine Institutional Review Board, all experiments were conducted in accordance with the Declaration of Helsinki and participants provided written informed consent.

### Resistance training and caloric restriction intervention descriptions

Details of the RT and CR interventions are published.^11^ Briefly, all participants in the study underwent 5 months of supervised RT 3 days per week on resistance machines. The machines used were: (1) leg press; (2) leg extension; (3) seated leg curl; (4) seated calf; (5) incline press; (6) compound row; (7) triceps press; and (8) bicep curl. The maximal weight that a person could lift with correct form in a single repetition (1RM) was used to prescribe intensity and the training goal was to complete three sets of 10 repetitions for each exercise at 70% 1RM for that specific exercise. Resistance was increased when a participant was able to complete 10 repetitions on the third set for two consecutive sessions. Strength testing was repeated every 4 weeks and training loads were adjusted to be consistent with the 70% 1RM goal. Each participant recorded the weight lifted, number of repetitions completed and number of sets completed for each exercise.

Participants in the RT+CR group performed the same RT program and also underwent CR for 5 months. The CR protocol used meal replacements (2 per day), nutrition education sessions (weekly) and dietary modification advice (as needed), targeting a 600 kcal per day reduction from estimated daily energy needs for weight maintenance. To verify compliance with the CR intervention protocol, participants were asked to keep a diet log of all foods consumed and the logs were monitored weekly by the study registered dietitian.

### Total body and thigh composition measures

Body composition measures were obtained at baseline, 5, and 23 months. Height and body mass were measured without shoes and outer garments removed. Body mass index was calculated as body mass (kg) divided by height squared (m^2^). Whole-body fat and lean mass (kg) were measured by dual-energy X-ray absorptiometry (DXA; Hologic Delphi QDR, Bedford, MA, USA) following standard procedures. Computed tomography (CT) scans, using a GE 16-slice Light Speed Pro (GE Medical Systems, Milwaukee, WI, USA), quantified total thigh volume, thigh muscle volume and thigh fat (total, subcutaneous and intermuscular) fat volume (cm^3^). Participants were placed supine in the scanner with arms above the head and legs flat. Scans were conducted at 120 KVp, 350 mA, 10-mm helical with a pitch of 11.25 mm per rotation and a gantry speed of 0.8 s. Thigh muscle and adipose tissue volumes were measured using a 5-cm section of the thigh centered at the junction of the proximal and middle-third of the femur as measured from the scout topogram. The volume of muscle and adipose tissue was segmented and measured using the GE Healthcare, Advantage Windows 4.2 Volume Viewer (GE Healthcare, Waukesha, WI, USA). Thigh muscle area was considered the total area of non-adipose and non-bone tissue within the deep fascial plane. For adipose tissue volume, sequences were reconstructed into the maximum 50 cm field-of-view to prevent truncation of subcutaneous fat on larger individuals and a 2.5-mm, no-gap, slice thickness was used. Intermuscular adipose tissue was separated from subcutaneous adipose tissue by drawing a line along the deep fascial plane surrounding the thigh muscles.

### Statistical analysis

Continuous body composition measures were summarized at each time point using means and standard deviations. Randomization group means at the 5- and 23-month assessment time points were modeled using mixed linear models fit with randomization, time and their interaction adjusted for baseline values of the outcome. Visit-specific group comparisons were performed using contrast statements at each follow-up visit. Absolute and percent changes in body weight, body composition and fat distribution variables were calculated as the baseline value subtracted from the 5-month value and 23-month value subtracted from 5-month value. Changes in body composition between baseline and 5 months and between 5 and 23 months were modeled using mixed linear models adjusted for baseline value of the outcome. Analyses were performed using the SAS software (version 9.4; SAS Institute, Cary, NC, USA) and *P*⩽0.05 was considered statistically significant.

## Results

The mean baseline age of all participants was 70±4 years, 50% were female (*n=*12) and the majority were non-Hispanic white (*n=*23). All participants were overweight or obese at baseline, with an average BMI of 29.9±2.0 kg m^−^^2^ (RT+CR=29.2±1.5 vs RT=30.7±2.3 kg m^−^^2^).

### Between- and within-group differences in total body mass and composition

Means (±s.d.) for total body mass and composition at baseline, at the end of the 5-month intervention and at the 23-month follow-up visit are presented by group in Table 1. RT and RT+CR groups were balanced at baseline with respect to total body mass and composition. RT+CR participants lost 7.1±2.4 kg of body mass (6.3±3.4% *P<*0.01) during the intervention, with ~74% of mass lost as fat mass and 26% as lean mass (both *P<*0.01). By design, total body mass and composition did not change significantly in the RT group during the 5-month study, and both total body fat and lean mass were significantly higher in the RT group compared with the RT+CR at the 5-month time point (both *P<*0.01). By 23 months, body mass and lean mass were still lower (*P<*0.01) in the RT+CR vs the RT group, but fat mass was not significantly different between groups.

### Between- and within-group differences in thigh composition

Thigh composition variables by group and time point are presented in Table 2, with groups balanced across all variables at baseline, save total thigh volume (1548.6±144.4 vs 1376.9±122.2 cm^3^ in the RT and RT+CR groups, respectively). Upon completion of the 5-month trial, all thigh compartment volumes were lower in the RT+CR group compared with the RT group. Total thigh fat volume (driven by decreases in all fat compartments) decreased in the RT+CR group (all *P*⩽0.01), whereas thigh muscle volume did not change during intervention in this group. In the RT group, thigh muscle volume increased by ~5% with the intervention, whereas thigh fat volumes were unchanged from baseline. Between-group 23-month comparisons showed no lasting intervention-related differences for any thigh composition measurements.

### Within-group changes in total body and thigh composition during postintervention follow-up

**Figure 1:**
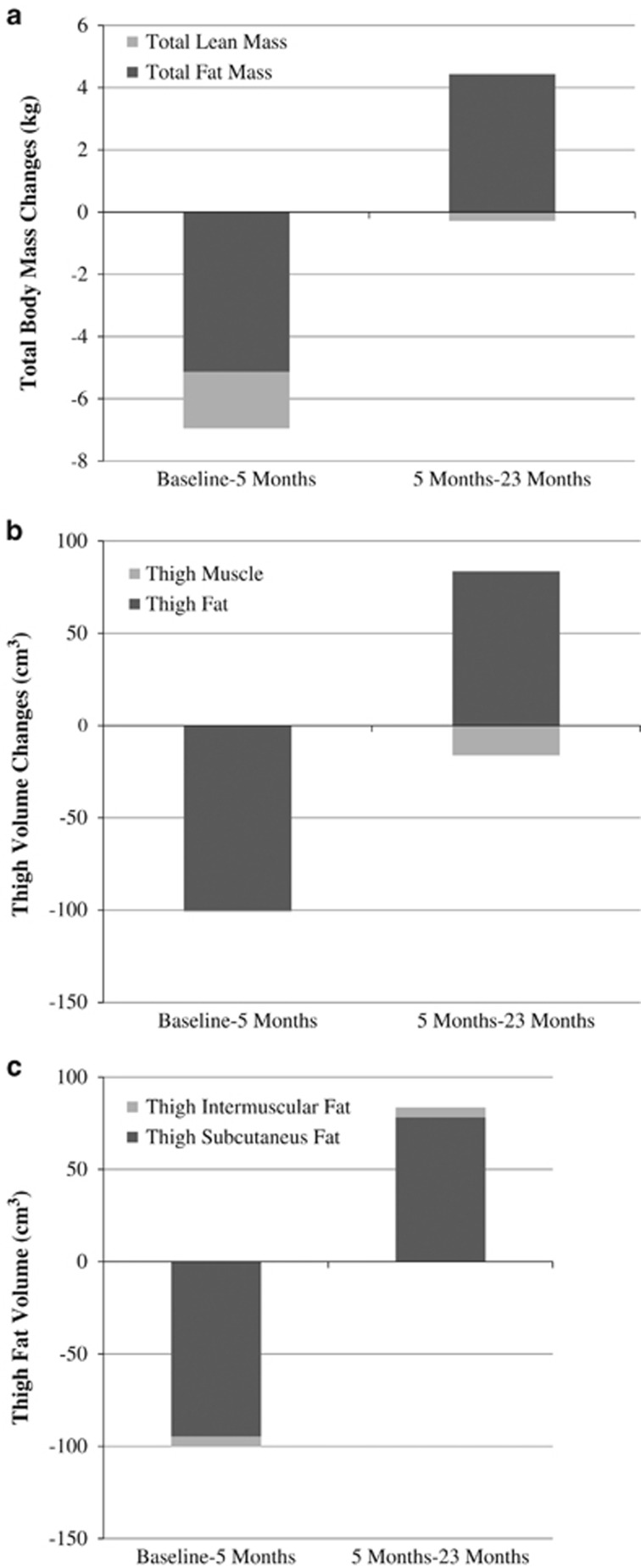
(**a**) Change in total fat and lean mass during and after the intervention period (RT+CR group, only). (**b**) Change in total thigh muscle and fat volume during and after the intervention period (RT+CR group, only). (**c**) Change in thigh subcutaneous and intermuscular fat volume during and after the intervention period (RT+CR group, only).

All RT+CR participants regained some body mass from 5 to 23 months (average: +4.8±2.6 kg; range: 0.4–9.1 kg); however, total body mass was still lower than baseline (83.9±11.5 vs 81.6±10.0 kg; *P<*0.01). In the RT group, body mass did not change during follow-up (−0.2±0.9 kg; *P=*0.87). Given the interest in studying the effect of weight regain *following intentional weight loss* on change in total body and thigh composition, Figures 1a–c present juxtaposed changes of the fat and lean compartments during and after the intervention for the RT+CR group. As shown in Figure 1a, less absolute fat mass was regained during follow-up (+4.4±0.6 kg from 5 to 23 months) than was lost during intervention (−5.1±0.3 kg from baseline to 5 months), whereas lean mass loss was observed both during and after the intervention (−1.8±0.4 kg from baseline to 5 months and −0.3±0.4 kg from 5 to 23 months) in RT+CR. Thus, a slightly greater percentage of the total mass that was regained was comprised of fat compared with the percentage of total mass that was lost as fat. There were no significant changes in fat or lean mass during months 5 to 23 in the RT-only group (see Table 2).

During the 18-month follow-up, RT+CR participants continued losing thigh muscle volume (−16.1±5.6 cm^3^) and regained thigh fat volume (+83.5±18.7 cm^3^; Figure 1b). Examination of changes in thigh intermuscular and subcutaneous fat volumes shows that most of the fat loss was from the subcutaneous compartment, and that ~82% of lost thigh subcutaneous fat was regained during the follow-up period (−94.7±16.7 cm^3^ from baseline to 5 months vs +78.1±18.5 cm^3^ from 5 to 23 months). Although a smaller amount of thigh intermuscular fat volume was lost with weight loss, nearly 100% was regained with weight regain (−5.5±1.6 cm^3^ from baseline to five months vs +5.4±1.4 cm^3^ from 5 to 23 months; Figure 1c). Thigh muscle volume, and thus total thigh volume, decreased during the 18-month follow-up in the RT-only group (both *P<*0.04), but there were no significant changes in any of the thigh fat volumes from months 5 to 23 (see Table 2).

## Discussion

The primary purpose of this study was to explore the legacy effects of a short-term RT+CR intervention, compared with RT alone that did not result in weight loss, on total body and thigh composition in overweight and obese, older adults. Our data indicate that despite clinically meaningful weight loss and concurrent favorable shifts in total body and thigh composition in participants who lost >5% of initial body mass by undergoing CR during RT, these improvements were generally not sustained long term. Indeed, at the 23-month follow-up visit, in spite of modestly reduced total body mass in the RT+CR group, total fat mass and thigh fat volumes (including intermuscular fat) were not different from participants who did not lose weight during RT alone. Furthermore, changes in body composition with weight regain during the follow-up show that there is total and regional fat accretion, but loss of lean tissue (particularly thigh muscle volume), implying greater long-term risk for sarcopenic obesity in those who lost weight vs those who did not during RT.

Observational cohort data in older adults examining body composition changes with weight loss and subsequent regain suggest that weight cycling may accelerate loss of lean mass with aging.^18, 19^ This finding is echoed in the few long-term follow-up studies of RCTs that resulted in weight loss, where weight regain following significant intentional weight loss is shown to be comprised predominantly of fat mass.^13, 24, 25^ For example, we previously showed that, in postmenopausal women, for every 1 kg of fat mass lost during weight loss, 0.26 kg of lean tissue was lost, whereas for every 1 kg of fat mass regained in the following year, only 0.12 kg of lean tissue was regained.^13^ Similarly, 30 months after completion of a 1-year weight loss intervention in frail, obese older adults, despite continued reduced total body mass from baseline, about one-half (45%) of total fat mass had been regained but lost lean mass was not recovered.^24^ Last, recent long-term data from the Look AHEAD trial examining changes in body composition over an 8-year follow-up period demonstrate that although a lifestyle-based caloric restriction and exercise program produced significant fat and lean mass loss during the first year of intervention, 7 years later, 100% of fat mass was regained, whereas participants continued to lose lean mass.^25^ However, none of these prior studies included a weight-stable control group in order to determine whether weight cycling is causal for these adverse changes in body composition. Our data show that, while both groups lost lean tissue during the postintervention follow-up period, the group that lost and regained weight (e.g., RT+CR) also experienced a gain in fat during this same period. Thus, these pilot data suggest that fat, rather than muscle, tissue is regained when older adults regain weight after weight loss. Over time, and/or with repeated bouts of weight cycling, this could exacerbate sarcopenic obesity in older adults.

Importantly, our results build on these existing published studies because, to the best of our knowledge, this is the first assessment of change in thigh composition with weight regain in older adults. These data may be even more clinically relevant as fat deposition in and around the thigh muscle (especially intermuscular fat) is an important body composition predictor of decline in gait speed, loss of muscle strength and onset of mobility disability among older adults.^5, 6, 7^ Group differences presented in this pilot study show that weight loss via CR during RT results in lower total, subcutaneous and intermuscular thigh fat, as well as lower thigh muscle volumes, compared with RT without weight loss immediately after intervention cessation. However, by 18 months postintervention, despite those who underwent caloric restriction maintaining a lower body mass, none of the group differences in thigh composition were evident; there was a trend (*P=*0.11) for muscle volume to still be higher in RT vs RT+CR. Notably, during the postintervention follow-up, RT+CR lost thigh muscle volume and partially regained total (81%) and subcutaneous (82%) thigh fat, but regained 100% of intermuscular fat. Thus, even though (on average) RT+CR participants did not regain all lost weight, fat mass or total thigh fat volume by 18 months, thigh muscle volume was lower and all of lost thigh intermuscular fat was regained.

Strengths of the current study consist of: (1) inclusion of a weight-stable control group, (2) long-term follow-up measures of total body and thigh composition using imaging techniques and (3) follow-up assessment of the subset of participants who lost a clinically significant amount of weight. Weaknesses include the potential selection bias which may have occurred because of our sampling methods. Because of the limited sample size and resources of this pilot project, a decision was made *a priori*, to ensure differential weight loss was achieved in RT+CR and RT groups. Although doing so prevents us from drawing conclusions regarding intent-to-treat effects of randomization to RT+CR or RT alone, we feel that the signal observed over time and between groups is strong enough to warrant replication in an appropriately powered RCT. Additionally, extrapolation of findings reported here should be limited to similar populations of healthy, but overweight and obese, adults in this age group. Findings may differ in older adults at more advanced ages and who may be frail or have more comorbidities.

In conclusion, despite clinically meaningful weight loss and concurrent favorable shifts in total body and thigh composition with short-term RT+CR, improvements do not appear to be sustained long-term relative to RT alone. Specifically, in the 18 months following the intervention, some amount of weight regain occurred in all RT+CR participants, reflected in fat mass and volume accretion, but thigh muscle volume loss. Replication of these findings are necessary and better understanding of the health correlates of weight regain in this population is warranted. Nonetheless, efforts to identify long-term weight loss maintenance strategies to prevent the predisposition for preferential total and regional fat accumulation with weight regain following intentional weight loss in older adults appear prudent.

## Figures and Tables

**Table 1:** Summary of total body mass and composition variables by group and time point

*Body composition variable*	*Group*	*BL*			*5 months*				*23 months*				
		*Mean*	*s.d.*	*Between-group* P*-value*	*Mean*	*s.d.*	*Δ from BL* P*-value*	*Between-group* P*-value*	*Mean*	*s.d.*	*Δ from BL* P*-value*	*Δ from 5 months*	*Between-group* P*-value*
Total body mass (kg)	RT	88.4	15	0.41	88.6	15	0.64	<0.01	88.5	15	0.9	0.86	0.03
	RT+CR	83.9	12		76.8	9.7	<0.01		81.6	10	0.01	<0.01	
Total body fat mass (kg)	RT	33.7	4.6	0.21	33.6	4.7	0.93	<0.01	34.4	5.4	0.28	0.24	0.16
	RT+CR	31.5	3.5		26.4	3	<0.01		30.8	3.5	0.41	<0.01	
Total body lean mass (kg)	RT	54.3	12	0.76	55.1	13	0.06	<0.01	54.4	12	0.95	0.13	<0.01
	RT+CR	52.9	10		51.1	9.7	<0.01		50.8	9.3	<0.01	0.49	

Sample sizes per group: RT=11, RT+CR=13. Within-group comparisons are estimated from mixed linear models adjusting for baseline mean values of the change variable from 0 to 5 and 5 to 23 months. Between-group comparisons estimated from mixed linear models adjusted for baseline mean values using contrasts for 5- and 23-month group means.

Abbreviations: BL, baseline; CR, caloric restriction; RT, resistance training.

**Table 2:** Thigh composition variables by group and time point

*Thigh composition variable*	*Group*	*BL*			*5 months*				*23 months*				
		*Mean*	*s.d.*	*Between-group* P*-value*	*Mean*	*s.d.*	*Δ from BL* P*-value*	*Between-group* P*-value*	*Mean*	*s.d.*	*Δ from BL* P*-value*	*Δ from 5 months* P*-value*	*Between-group* P*-value*
Total thigh volume (cm^3^)	RT	1549	144	0.01	1557	133	0.77	<0.01	1494	136	0.08	0.02	0.2
	RT+CR	1377	122		1289	88.6	<0.01		1330	120	0.16	0.19	
Thigh muscle volume (cm^3^)	RT	664.4	140	0.85	695.8	166	0.01	0.01	671.1	154	0.61	0.04	0.11
	RT+CR	653.4	140		652.7	142	0.9		636.7	129	0.03	0.01	
Thigh fat volume (cm^3^)	RT	763.2	208	0.13	763.2	240	0.99	<0.01	751.4	209	0.57	0.62	0.59
	RT+CR	634.2	197		534	152	<0.01		617.5	180	0.48	<0.01	
Thigh subcutaneous fat volume (cm^3^)	RT	732.5	209	0.16	733.7	241	0.9	<0.01	720.1	213	0.53	0.56	0.65
	RT+CR	609.6	199		514.9	153	<0.01		593	183	0.48	<0.01	
Thigh Intermuscular Fat Volume (cm^3^)	RT	30.7	19.7	0.32	29.5	16.2	0.37	0.01	31.3	22.7	0.82	0.47	0.53
	RT+CR	24.6	8		19.1	6.9	<0.01		24.5	9.6	0.95	<0.01	

Sample sizes per group: RT=11, RT+CR=13. Within-group comparisons are estimated from mixed linear models adjusting for baseline mean values of the change variable from 0 to 5 and 5 to 23 months. Between-group comparisons estimated from mixed linear models adjusted for baseline mean values using contrasts for 5- and 23-month group means.

Abbreviations: BL, baseline; CR, caloric restriction; RT, resistance training.
